# A QR Code–Based Contact Tracing Framework for Sustainable Containment of COVID-19: Evaluation of an Approach to Assist the Return to Normal Activity

**DOI:** 10.2196/22321

**Published:** 2020-09-07

**Authors:** Ichiro Nakamoto, Sheng Wang, Yan Guo, Weiqing Zhuang

**Affiliations:** 1 School of Internet Economics and Business Fujian University of Technology Fuzhou China

**Keywords:** COVID-19, coronavirus, symptom-based, quick response, eHealth, digital health, telesurveillance, pandemic, epidemic, interoperability

## Abstract

We discuss a pandemic management framework using symptom-based quick response (QR) codes to contain the spread of COVID-19. In this approach, symptom-based QR health codes are issued by public health authorities. The codes do not retrieve the location data of the users; instead, two different colors are displayed to differentiate the health status of individuals. The QR codes are officially regarded as electronic certificates of individuals’ health status, and can be used for contact tracing, exposure risk self-triage, self-update of health status, health care appointments, and contact-free psychiatric consultations. This approach can be effectively deployed as a uniform platform interconnecting a variety of responders (eg, individuals, institutions, and public authorities) who are affected by the pandemic, which minimizes the errors of manual operation and the costs of fragmented coordination. At the same time, this approach enhances the promptness, interoperability, credibility, and traceability of containment measures. The proposed approach not only provides a supplemental mechanism for manual control measures but also addresses the partial failures of pandemic management tools in the abovementioned facets. The QR tool has been formally deployed in Fujian, a province located in southeast China that has a population of nearly 40 million people. All individuals aged ≥3 years were officially requested to present their QR code during daily public activities, such as when using public transportation systems, working at institutions, and entering or exiting schools. The deployment of this approach has achieved sizeable containment effects and played remarkable roles in shifting the negative gross domestic product (–6.8%) to a positive value by July 2020. The number of cumulative patients with COVID-19 in this setting was confined to 363, of whom 361 had recovered (recovery rate 99.4%) as of July 12, 2020. A simulation showed that if only partial measures of the framework were followed, the number of cumulative cases of COVID-19 could potentially increase ten-fold. This approach can serve as a reliable solution to counteract the emergency of a public health crisis; as a routine tool to enhance the level of public health; to accelerate the recovery of social activities; to assist decision making for policy makers; and as a sustainable measure that enables scalability.

## Introduction

COVID-19, which is caused by SARS-CoV-2, has resulted in millions of confirmed cases and hundreds of thousands of deaths worldwide since the first cases were officially reported in December 2019; it has been declared a global pandemic by the World Health Organization (WHO) [1]. The negative impacts of COVID-19 are unprecedented and pervasive at a variety of levels, extensively spreading from individuals and institutions to public authorities. For individuals, COVID-19 is not only a crisis of physical health but has substantially affected mental health, as evidenced by the growing number of consultations regarding psychological stress, anxiety, and depression [2]. Prior research shows that the percentages of the population who are concerned about symptoms, prevention and therapy, and psychological problems during the COVID-19 epidemic are 65%, 15%, and 11%, respectively; these levels are commensurate with those of the past outbreak of severe acute respiratory syndrome (SARS) [3,4]. Further, the pandemic has caused remarkable decreases in the gross domestic product (GDP) in many economies; thus, firms must employ measures to resume production and survive unexpected disasters in the future [5]. In the absence of credible and traceable information, it is difficult for governments to make coherent and accurate decisions [6]. Lifting the lockdown of COVID-19 is an enormous challenge because in the absence of effective screening and isolation aided by digital health, the second wave outbreak of COVID-19 cannot be precisely predicted contingent on present scientific understanding [5,7]. Subclinical infection through asymptomatic and presymptomatic transmission unnoticeable to individuals in close contact and delays in sharing of knowledge complicate the trajectory of the outbreak; therefore, the pandemic will take a longer time to subside [4,8]. The curve of the first wave of the outbreak may flatten, followed by a sizeable reduction in new infections, which will promote the opportunity to reopen the economy [8,9]. However, even if workplaces resume their interrupted production, doubts will be cast over whether it is feasible to sustain the same pace that was employed prior to the outbreak. Given the infectiousness of COVID-19 and the dynamics of high subclinical transmission, controlling the epidemic by mere manual contact tracing is less effective and infeasible. The WHO has forecast that the pandemic is still in its early stage, which necessitates long-term efforts in combating COVID-19. Hence, solutions to these challenges are of concern to individuals, firms, and public authorities that are struggling to return to normal rhythms [10].

A range of digital health approaches have been employed to contain the spread of disease during the current COVID-19 pandemic and past pandemics [11-20]. These control measures have been proved to be effective for numerous countries in productively depleting the first wave of COVID-19; among these measures, contact tracing is considered to be the centerpiece of containment, and it attracts a great deal of attention. Contact tracing involves identifying, quarantining, and alerting contacts of infected individuals [13]. Some countries that ceased contact tracing due to high prevalence of COVID-19 are currently reinstituting this strategy to curtail the potential impact of the second wave of the outbreak [11]. However, contentious issues facing contact tracing apps include the debate on which deployment framework (ie, centralized versus decentralized) and sensor technology (ie, Bluetooth, GPS, or quick response [QR]) can better address critical challenges such as effectiveness and sustainability of containment. Centralized architecture mostly complies with the Pan-European Privacy-Preserving Proximity Tracing (PEPP-PT) protocol, and personal data is collected for public control. In contrast, the decentralized approach mainly follows the Decentralized Privacy-Preserving Proximity Tracing (DP-3T) protocol; consequently, private information is not collected, and inference of exposure is constrained only to local devices [16]. However, even in the decentralized framework, a centralized database that at least accommodates the infected is of necessity to guarantee reliability. Hence, essentially, the concept of decentralization is only partially tenable [14-16]. Decentralized systems mostly do not retrieve sensitive data of users such as locations, and sharing permission is requested to preserve the users’ privacy. These systems hinge substantially on peer-to-peer self-reporting that is trustworthy by the peer network; thus, high participation of the population is essential for meaningful containment. This mechanism may not completely address all likely routes of transmission between individuals even with full participation, accounting for potential delay in knowledge sharing [16]. The decentralized Bluetooth framework recently coprovided by Apple and Google supports preservation of privacy and provides users with anonymized contact exposure and guidance on how to respond if a risk is identified. However, because the nonidentifiable data are either changed or deleted periodically, traceability of the outbreak is jeopardized. Second, the definition of close contact is rather flexible; therefore, the detection precision of exposure may differ in different settings. Third, globally, only approximately one-quarter of smartphones are compatible with the Bluetooth standard required by Google and Apple, which inevitably inhibits the effectiveness of contact tracing [14-16]. The latest rebounding of confirmed cases in Japan has demonstrated the fragility of the Bluetooth framework [1]. On the other hand, extant contact tracing apps that collect GPS data to identify exposure improve the credibility of data. This supports evidence-based decision making but raises other concerns. The first concern is that location is a close proxy for contact but is not equal to it; therefore, the contact inference based on GPS data could generate confusion for the public due to bias in technical precision. The second concern is how to curb misuse and unauthorized access to sensitive information [16,19].

Beginning with contact tracing, telesurveillance has become more sophisticated, covering technologies from telecare to triage (eg, sorting of patients). Although the aforementioned tools and techniques may remarkably enhance the current capabilities of containment and reduce the spread of COVID-19, a broader framework is needed to increase its effectiveness through the integration of existing digital health measures [12,13,16,17]. The negative impacts of COVID-19 on physical health, mental health, and social rhythms are dramatic and far-reaching; hence, individuals, institutions, and public authorities urgently need integrated guidance and delay-free information that can safeguard the well-being of the public and assist them in making a smooth transition back to work and normal activities [18]. The lack of an inclusive repository with a uniform structure causes repeated failures for extant telesurveillance. Compared with the GPS-based approach, symptom-based QR contact tracing does not identify the location details of users; thus, the vulnerability inherent to the GPS-based approach can be waived. Further, credibility and traceability can be enhanced relative to the self-report mechanism used in the Bluetooth approach [16,18,19].

In contrast to location-coupled QR contact tracing, here, we evaluate a symptom-based QR framework that addresses the disadvantages of GPS and Bluetooth. The major aim of this paper is to appraise how this approach can serve as an urgent countermeasure against the COVID-19 crisis as well as a routine tool to guide the return of daily activities in workplaces, travel locations, and communities to normal rhythms.

## Core Concept and Framework of the Tool

### Features for Individuals

**Figure 1:**
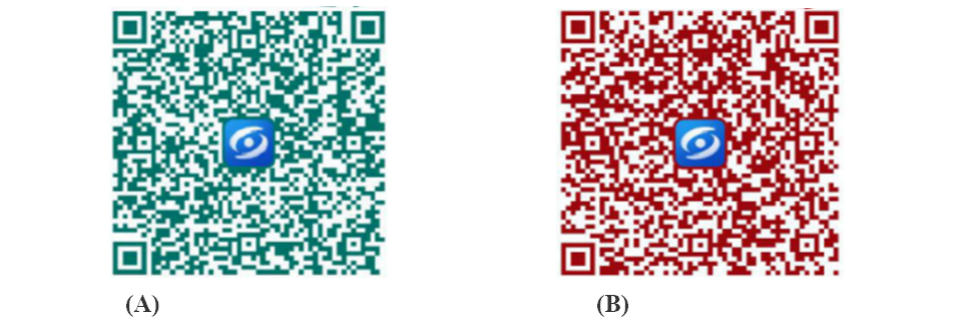
Color schema of the quick response codes: (A) green; (B) orange.

The first crucial concept used in the approach is symptom-based QR health codes issued by public health authorities. The codes do not retrieve the location data of the users; instead, two colors are supported to differentiate the health status of individuals. The QR codes are illustrated in Figure 1. A green code denotes that the individual is not infected with COVID-19 according to a polymerase chain reaction (PCR) test record; thus, the individual passes the health verification test. In contrast, an orange code signals that the person is either infected with COVID-19 or the likelihood of being infected is high. This can be stratified into six scenarios in which the individual:

1. Is infected with COVID-19
2. Had close contact with an individual who is infected with COVID-19
3. Comes from a region where the infection rate of COVID-19 is high
4. Is a resident of a community under strict public surveillance due to severe infection
5. Has a record of a high fever within the past 14 days
6. Has a record indicating the purchase of anti-fever medicine within the past 14 days

The QR codes are officially regarded as electronic certificates of individuals’ health status. The information in the codes is automatically read and analyzed by QR scanners. The core notions of the design are to enhance the credibility of data, increase the speed of processing, and reduce errors arising from manual operation.

The second crucial concept is the synthesis of critical features, including contact tracing, exposure risk self-triage, self-update of health status, health care appointments, contact-free psychiatric consultation, and QR codes for other family members. The platform also coherently merges health insurance and prescription services, which reduces the costs and delays of operation for users.

### Features for Institutions

The QR approach consolidates features that can assist companies and institutions with early and automatic case screening and flexible social distancing based on QR information. This avoids crosstransmission between workers and guarantees smooth resumption of production at the normal rhythms. Each worker can double-check the health colors of their colleagues (no other private information is shared), which lowers systematic mistakes of mis-surveillance. The accounts of the QR scanning sites are configurable and scalable. Firms can choose to report local statistics to public authorities for subsequent analysis. Coupled with proximity detection techniques, an alarm can be raised in scenarios of potential close contact. Managers can thus establish a flexible and correctable threshold to implement automatic reminders of social distancing as science gains updated knowledge of the disease.

### Features for Public Authorities

The QR approach can help policy makers make more effective decisions and improve public surveillance. The accounts of QR scanning sites at travel spots are administered by public authorities. Any individual must present their QR code to use public transportation systems such as buses, railways, or airports. The summary at each site is synchronized with public authorities for general analysis.

**Figure 2:**
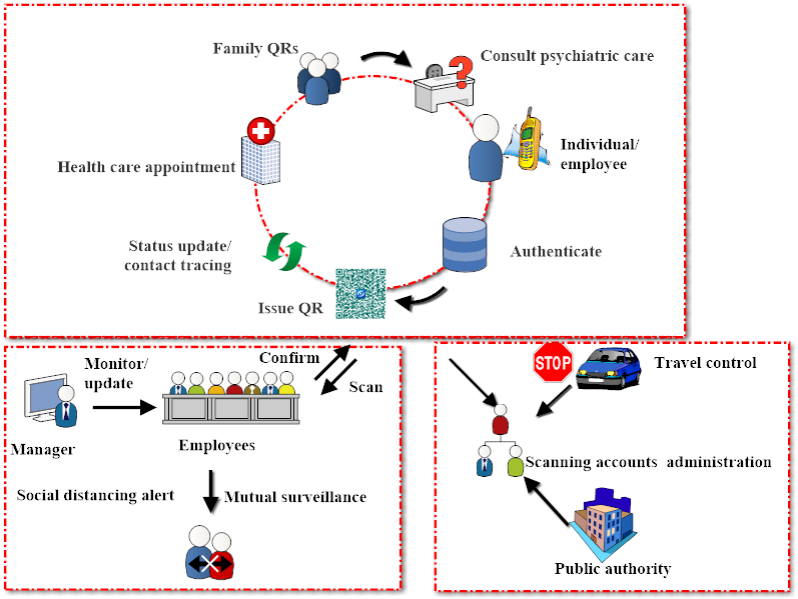
Diagram of the QR-based approach integrating features critical to individuals, institutions, and public authorities. QR: quick response.

**Figure 3:**
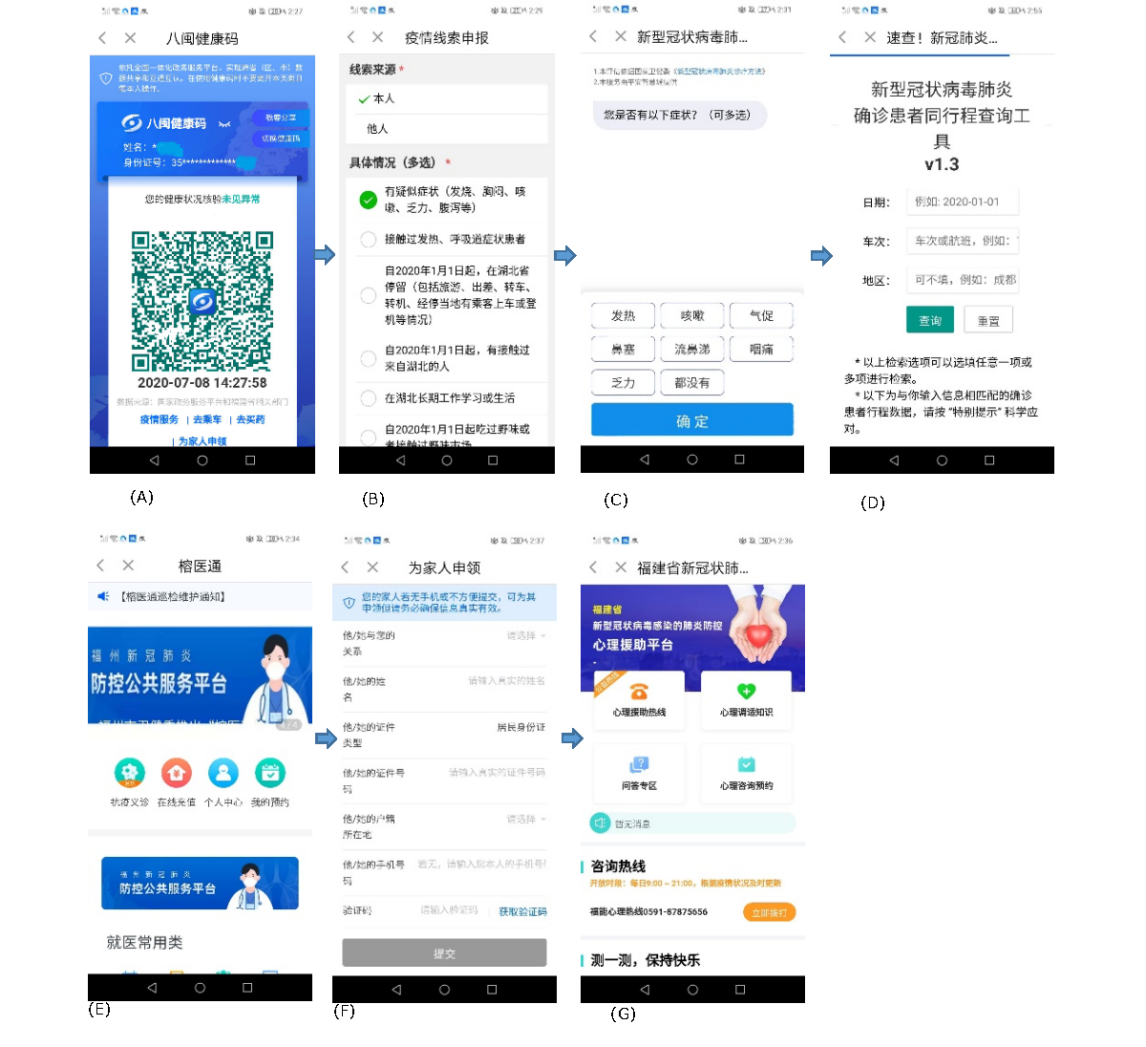
Screenshots of the QR-based approach for individuals: (A) QR code, (B) health status update, (C) risk self-triage, (D) contact tracing, (E) health care appointment, (F) request for QR codes for family members, (G) psychiatric consultation.

The QR-based uniform design increases the seamless synergy among individuals, institutions, and public authorities and reduces delays in information processing and transmission. In Table 1, we illustrate the core concepts and potential scenarios of implementation of the QR approach. Figures 2 and 3 present a brief diagram and screenshots of the approach, respectively.

**Table 1:** Concepts of the QR-based framework.

Concept and subject		Description
**Products**		
	Individuals	Mobile apps (iOS and Android)
	Institutions and public authorities	Web-based platforms (Windows)
**Scenarios**		
	Individuals	Electronic certificates of health status
		Dynamic of updates health status
		Self-triage of exposure risk and self-isolation
		Contact tracing of past potential exposure
		Noncontact health care appointments when infected or suspected of being infected
		QR^a^ codes for other family members
		Remote consultations on psychiatric care
	Institutions	Surveillance of the health status of employees and close contacts
		Setup of automatic social distancing alerts
		Mutual surveillance between employees
	Public authorities	Issuance and administration of QR codes
		QR-based travel control
		QR code scanning account management
		Statistics and outbreak inference
**Strengths**		
	Participation	Equity of access and high participation
	Cost	Reduced costs of manual operation and cross-platform synergy
	Errors	Reduced errors of manual operation
	Other	Sustainability and scalability

^a^QR: quick response.

## Quantitative Analysis of the Effectiveness of the Approach

To apply a cross-platform surveillance approach and to determine the best way to integrate discrete components into a uniform health system, evidence is required of the effectiveness of the approach in practice, with particular scrutiny of the context in which it is to be deployed [3].

Since the spread of COVID-19 began, the QR tool has been formally deployed in Fujian, a province located in southeast China that had a population of nearly 40 million people by late June 2020. All individuals aged ≥3 years were officially requested to present their QR code during daily public activities, such as when using public transportation systems, working at institutions, and entering or exiting schools. Almost 5 million individuals left Wuhan, the epicenter of the COVID-19 pandemic in China, when the outbreak occurred. Approximately one-third of these individuals traveled to other regions, such as Fujian Province [21]. With the app in use, by July 12, 2020, the cumulative positive cases of COVID-19 were confined to 363 individuals, of which 361 (99.4%) recovered from the disease. Only one patient died and one patient remained positive for COVID-19 at that point. Meaningfully effective containment was achieved through a strict strategy of centralized control and extensive deployment of the tool. The latest data show that since the outbreak started, the GDP of provinces in China, including Fujian, decreased to –6.8% in the first quarter of 2020 due to the nationwide lockdown. However, due to the gradual lifting of lockdown and reopening of production, the GDP bounced back and became positive by July [5].

**Figure 4:**
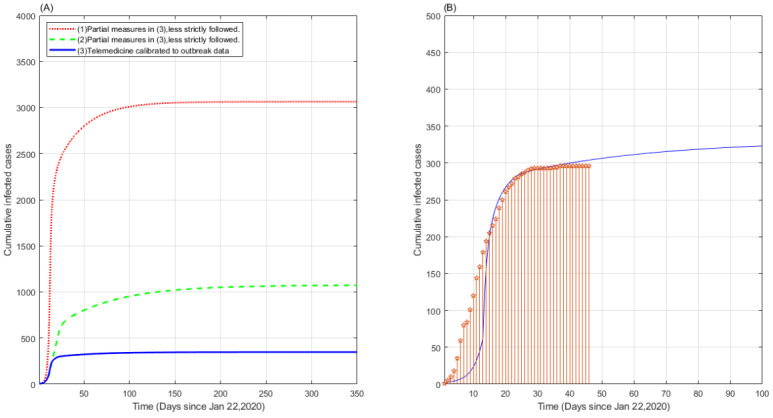
Simulation of heterogeneous countermeasures during the COVID-19 outbreak. (A) Model simulation of different scenarios; (B) comparison of the model simulation with the observed data.

As shown in Figure 4, we deployed the model introduced in [22] to simulate the heterogeneous epidemic evolution of the COVID-19 outbreak in Fujian using data published by Johns Hopkins University [23]. The epidemiological mean-field framework sketched in [22] captures the average effect involving the whole population, which extends the classical susceptible-infected- recovered (SIR) model and evaluates more complicated disease transmission scenarios partitioned into eight stages of infection, including susceptible, infected, diagnosed, ailing, recognized, threatened, healed, and died. The status of each stage is determined by the interactions among different adjacent stages. The core concept hinges on the deliberation that subdivided models enhance the accuracy of portraying the dynamic spread of COVID-19. This model estimates how progressive countermeasures would affect the spread of the pandemic. The system has been shown to correctly delineate the dynamics of the epidemic and is suitable for the prediction of containment measures with varying strengths and natures. The simulation denotes that restrictive social distancing measures must be effectively combined with contact tracing and other countermeasures to decrease the spread of COVID-19. The assumptions of the model are that the stages of infection are mutually exclusive; the likelihood of being susceptible again after recovering from the infection is negligible; there is a distinction between undiagnosed and diagnosed individuals; and there is a delay in the emergence of symptoms.

We calibrated the model to the Fujian outbreak data starting from January 22, 2020, when the first case was identified there (see Figure 4(A), blue solid curve, and Figure 4(B)). The curves in Figure 4(A) denote the cases where the features of digital health in our framework were adopted in chronological order to counteract the spread of disease up to a 1-year horizon. The imposed countermeasures are as follows: (1) social distancing, including in workplaces, and mutual surveillance between workers is followed from day 2; (2) contact tracing of individuals in close contact with infected people is appended starting on day 12; (3) contact tracing of individuals with a record of high fever or record of the purchase of anti-fever medicine is added on day 22; (4) contact-free psychiatric consultations and travel control measures are followed. Comparatively, these measures can be employed simultaneously in practice. The blue solid curve (Figure 4(A)) corresponds to the approach using all the above four measures. The simulated cumulative cases amounted to nearly 350 cases by July 2020, which is close to the observed outbreak data in practice. The red dotted curve indicates the cases where (1) and (2) are followed but less strictly, after which the number of cumulative cases would increase almost ten-fold to 3100 half a year later. In contrast, the green dashed curve implies the case where measures (2), (3), and (4) are more loosely followed. This scenario caused about three-fold growth in the number of cumulative cases to 1100 cases by July. Hence, the strict deployment of the merged measures remarkably contained the spread of COVID-19.

## Strengths and Limitations of the Approach

The centralized approach is distinct from other decentralized models in three paramount ways. The first is the integration of features that are important to the concerned population in one identical platform; this is pivotal when combating highly contagious diseases, as delays of data sharing can enable increased spread of COVID-19. Tools used at the individual or public authority level may not respond in a timely and accurate manner to requirements at the institutional level. The collective provision of features oriented toward a wider range of users promotes broader applicability of the platform. In lieu of the spontaneous self-reporting that is mostly used in decentralized frameworks, where the agreement of individuals is requested, this approach affirms high, wait-free participation. As delays and misinformation are still of public concern, this responds to the urgent need of a uniform framework internalizing various sources of corroboration and efforts. Second, the tool facilitates self-triage for individuals and self-scheduling for institutions. Hence, load balancing of the pressure on overburdened health care systems is plausible. The tool also prevents unnecessary and risky in-person visits. Third, due to its interoperability, credibility, and traceability, the QR approach can play valuable roles as both an emergent and routine tool to counteract COVID-19.

Several concerns must be addressed to ensure successful implementation of this tool. First, it is important to prevent malicious or unauthorized use of the QR data. Second, for economic settings where the general population is highly sensitive to preservation of privacy, acceptance of this approach will be compromised; thus, the effects of containment must be closely observed.

Illegal or unauthorized use of health care information is detrimental at a variety of levels [24]. Reports forecast that misuse of this information will cause an average financial loss of nearly US $7.13 million worldwide in 2020 [9]. Unlawful use of data can damage the reputation of service providers and negatively impact the confidence and health of patients. The Harris study targeting 1527 individuals found that 123/1527 (8.1%) of the respondents refrained from participating in health programs due to reduced confidence in the quality of data protection [25]. Another study conducted with 3959 individuals suggested that unauthorized use of patients’ information would increase the likelihood of negative perceptions and responses. It was found that almost two-thirds of the participants (2764/3959, 69.8%) expressed security-related concerns, which impeded subsequent health care interventions and interactions [26]. A simulation performed at Oxford University implied that digital contact tracing interventions would lose the potential to substantially contain the spread of COVID-19 if the population uptake rate decreased approximately below 60%, even if these interventions were deliberately implemented alongside other countermeasures [27].

## Conclusions

The framework presented in this study has substantially controlled the spread of COVID-19 in Fujian Province, where almost 40 million people reside, since the first case was officially reported in January 2020. Of the 363 reported cumulative cases, 361 (99.4%), recovered, 1 patient died (mortality rate 0.3%) and one patient remained positive for COVID-19 (0.3%) as of July 12, 2020. Due to the early deployment and rigid implementation of the approach, it successfully helped transition the GDP from –6.8% to a positive value by July. Firms have succeeded in gradually resuming normal production without sacrificing effective containment. The tool has assisted public authorities with effective containment and travel control. The model simulation shows that if the partial measures were followed less rigorously, the likelihood of confirmed cases would increase, leading to multiple times of growth in the number of cumulative cases of COVID-19. Due to the credibility, interoperability, and sustainability empowered by the design, long-term containment of COVID-19 is feasible. Digital health is a principal factor contributing to the success of containment; however, it cannot solve all the challenges the world is presently facing [3]. The convergence of COVID-19 diagnostics and treatment provides opportunities to deliver potentially disruptive technologies to drive the development of integrated health systems. These systems should increase accessibility to contain the pandemic while improving the promptness, interoperability, and credibility of outbreak detection and surveillance while guiding more precise and sustainable public health responses [28].

The deployment of telesurveillance to specific settings must account for both technical and nontechnical factors. The effectiveness of telesurveillance may vary due to cultural conflicts as well as users’ moral and religious backgrounds in different countries and regions [29]. The collaboration of stratified participants should be guided by law enforcement for better protection of individuals’ data, preventing malicious breaches of privacy information as well as abuse beyond the scope of legal screening, contact tracing, and surveillance [30]. This symptom-based QR approach facilitates the optimized allocation of limited health care resources [28,31]. It clearly aids the identification and isolation of cases at an earlier stage and generates seamless, delay-free cooperation of individuals, institutions, public authorities, and other responders in both the short term and in the long term. The approach can be used both to effectively counteract the emergency of a public health crisis and as a routine surveillance technique in the postpandemic era to facilitate rapid recovery from the shock of the outbreak. The integration of features that are critical for the containment of COVID-19 in a uniform platform will be a research trend to achieve more effective control. The capabilities of rapid response, traceability, and credibility provided by this approach can help society to achieve a balance between sustainable containment and smooth recovery of the economy. This tool is scalable for extension of functionality with advances in artificial intelligence, big data, and other technologies. It will enable coordinated data-sharing mechanisms ahead of, during, and after an epidemic, improving the quality and sustainability of data in an unprecedented era of high-impact and cyclical pandemics. Information obtained from the app can also increase scientific understanding of the dynamics of COVID-19 and deliver positive insight for other infected communities.

## Abbreviations

DP-3T: Decentralized Privacy-Preserving Proximity Tracing
GDP: gross domestic product
PCR: polymerase chain reaction
PEPP-PT: Pan-European Privacy-Preserving Proximity Tracing
QR: quick response
SARS: severe acute respiratory syndrome
SIR: susceptible-infected-recovered
WHO: World Health Organization
